# Efficacy and Safety of Central Memory T Cells Combined With Adjuvant Therapy to Prevent Recurrence of Hepatocellular Carcinoma With Microvascular Invasion: A Pilot Study

**DOI:** 10.3389/fonc.2021.781029

**Published:** 2021-12-03

**Authors:** Jianqiang Cai, Jianjun Zhao, Defang Liu, Huangfan Xie, Hailong Qi, Junfan Ma, Zhongjie Sun, Hong Zhao

**Affiliations:** ^1^ Department of Hepatobiliary Surgery, State Key Laboratory of Molecular Oncology, National Cancer Center/National Clinical Research Center for Cancer/Cancer Hospital, Chinese Academy of Medical Sciences and Peking Union Medical College, Beijing, China; ^2^ Department of New Drug Registration, Hebei Immune Cell Application Engineering Research Center/Baoding Newish Technology Co., LTD/Newish Technology (Beijing) Co., LTD, Beijing, China; ^3^ School of Pharmaceutical Sciences, Tsinghua University, Beijing, China; ^4^ State Key Laboratory of Elemento-Organic Chemistry, College of Chemistry, Nankai University, Tianjin, China

**Keywords:** hepatocellular carcinoma, microvascular invasion, adjuvant therapy, Tcm treatment, clinical study

## Abstract

**Background:**

Postoperative adjuvant transcatheter arterial chemoembolization (TACE) following curative hepatectomy has been reported to improve the clinical outcomes of hepatocellular carcinoma (HCC) patients with microvascular invasion (MVI), but more endeavors are required to achieve greater clinical benefit. Central memory T-cell (Tcm) self-transfusion has shown superior antitumor activity in several preclinical studies; however, clinical studies are rare. The aim of this study was to evaluate the clinical benefit and safety of combination treatment with Tcm self-transfusion and TACE as adjuvant treatment in HCC patients with MVI after curative hepatectomy.

**Methods:**

From October 2016 to September 2018, primary HCC patients with histologically confirmed MVI who underwent curative hepatectomy at the Cancer Hospital of the Chinese Academy of Medical Sciences were recruited for this study. The patients were divided into a Tcm group (combined Tcm self-transfusion with TACE treatment) or a control group (TACE treatment alone) according to their willingness. The recurrence-free survival (RFS), quality-of-life (QOL) score, and adverse events of each patient were recorded within 2 years.

**Results:**

A total of 52 patients were enrolled, and 48 were eligible for the final data analysis. The median follow-up time was 20.5 months (95% CI: 17.05–22.55 months). The median RFS time was 9.5 months in the control group; the cutoff date was not reached in the Tcm group (when the follow-up duration was 12 months, *p* = 0.049, HR = 0.40; 95% CI: 0.16–0.99). Compared with the control group, 1- and 2-year RFS rates were higher in the Tcm group (72.0% *vs.* 46.4% and 58.18% *vs.* 39.14%, respectively). Multivariate analysis did not indicate that Tcm treatment was an independent prognostic factor associated with HCC recurrence (*p* = 0.107, HR = 2.312; 95% CI: 0.835–6.400), which might be due to the small sample size of this study. Nevertheless, Tcm treatment effectively improved a reduced QOL due to HCC and liver function injury. Finally, the safety profile of Tcm treatment in this study was good, without any serious adverse events.

**Conclusions:**

This pilot study showed that Tcm self-transfusion combined with TACE treatment might be a beneficial adjuvant therapy with good safety for primary HCC patients with MVI after curative hepatectomy.

**Trial registration number:**

NCT03575806

## Introduction

Hepatocellular carcinoma (HCC) accounts for approximately 90% of liver cancer cases and is the sixth most common cancer and fourth leading cause of cancer-related death worldwide, with an estimated 841,080 new cases and 781,631 deaths in 2018 (1, 2). The highest incidence and mortality rates of HCC are reported in Eastern Asia and sub-Saharan Africa, where the main risk factor is cirrhosis caused by hepatitis B virus (HBV) or hepatitis C virus (HCV) infection (2). Currently, hepatectomy is one of the most reliable therapies for HCC (3, 4), though, high postoperative recurrence remains a serious problem (5). In China, the 5-year survival rate for liver cancer is only 12.1%. The rate of tumor recurrence and metastasis 5 years after hepatocellular carcinoma resection is as high as 40%–70%; approximately 50% of HCC patients experience recurrence within 2 years (6–8). Yet, there is no recognized drug or treatment to prevent recurrence of liver cancer in the world at present.

Several major risk factors have been identified as being closely linked to the postoperative recurrence of HCC, including blood vessel invasion, the number of nodules, tumor size, preoperative glutamic oxaloacetic aminotransferase (AST) elevation, resection margin, and liver capsule invasion; blood vessel invasion includes macrovascular invasion/portal vein tumor thrombosis (PVTT) and microvascular invasion (MVI) (3, 7, 9–11). MVI is defined as invasion of the intrahepatic portal vein or hepatic vein branches by tumor cells; the incidence rate of MVI is 15%~57.1% in HCC patients, and MVI indicates aggressive tumor behavior, with a greater tumor burden (12–14). MVI is generally considered an independent risk factor for intrahepatic metastasis and early tumor recurrence (15, 16). It has been demonstrated that the degree of MVI described by invaded vessels, invading carcinoma cells, and the distance of invasion from the tumor edge is valuable for predicting prognosis after curative hepatectomy (17, 18). Statistically, the time of recurrence-free survival (RFS) in HCC patients with MVI is shorter than that in HCC patients without blood vessel invasion (19). As there is no established standard adjuvant treatment for HCC patients with MVI following hepatectomy, developing effective modalities to prevent the postoperative recurrence of HCC in patients with MVI is of great significance (20).

Current treatments to prevent postoperative recurrence in patients with HCC with MVI include postoperative adjuvant transarterial chemoembolization (pa-TACE), postoperative radiotherapy, radiofrequency ablation (RFA), and sorafenib, and pa-TACE is the most common adjuvant therapy after curative resection (21). Through arterial injection of chemotherapeutic drugs and embolizing agents, TACE decreases blood flow to the tumor and induces tumor ischemic necrosis (22). Many studies have reported that pa-TACE improves the overall survival (OS) and RFS of HCC patients with blood vessel invasion after curative hepatectomy (20, 23–26). However, due to controversial reports on the clinical benefits of pa-TACE (27, 28), the optimal postoperative adjuvant treatment for preventing HCC recurrence in patients with MVI reaches no consensus and requires further investigation. Thus, it remains an area where clinical needs are not being met. It is urgent to conduct clinical trials to evaluate the efficacy of adjuvant therapy for patients with high risk of recurrence after radical HCC surgery, which has become the primary task in the postoperative treatment of HCC.

Recently, immunotherapy for cancer treatment has gained growing attention, and adoptive cell transfer of immune effector cells has been shown to have clinical benefit in postoperative HCC patients (29, 30). For instance, a meta-analysis including 11 clinical studies reported that dendritic cell and cytokine-induced killer cell (DC-CIK cell) transfusion combined with TACE/TACE+RFA markedly improved RFS and OS compared with simple TACE/TACE+RFA (31). Moreover, a phase 3 randomized controlled trial in Korea demonstrated that postoperative adjuvant transfusion of active CIK cells notably increased the RFS and OS of HCC patients (32, 33), with efficacy lasting for more than 5 years (34). Despite these positive results, the clinical outcomes of CIK cell-based immunotherapy remain controversial, and its effect in postoperative HCC patients with MVI has not been exclusively studied (35, 36). Other immune cells with high cytotoxicity, such as tumor-infiltrating lymphocytes and peripheral blood T cells (37, 38), might be ideal sources for adoptive cell therapy to prevent postoperative recurrence in HCC patients with MVI.

In this study, we focused on central memory T cells (Tcms), which can quickly differentiate and proliferate into effector T cells, extensively secrete effector cytokines, and strongly activate the memory immune response when again encountering the same antigen (39, 40). Different from effector T cells, memory T cells express CD45RO and can be divided into two populations according to the expression of the lymphoid-homing molecule CD62L, with Tcms being CD62L positive and effector memory T cells (Tems) CD62L negative (41). For this reason, Tcms home to and reside in lymph nodes, while Tems reside in blood, spleen, and peripheral tissues. Tcms have a long life and can rapidly proliferate and differentiate upon secondary response, thus possess superior immune activity than Tems and effector T cells in immune therapy. Using Tem transfusion as a control, Tcm transfusion together with tumor vaccination effectively inhibits tumor growth in a mouse xenograft melanoma model (42), and mesothelin-specific Tcm infusion significantly extends the survival of mesothelioma-bearing NSG mice (43). Furthermore, Busch et al. found that successive transfer of a single Tcm was able to rebuild the lymphoid population and phenotypical diversity of T cells after homing to lymphoid or nonlymphoid organs; moreover, Tcm descendants ultimately reconstituted immunocompetence against lethal infection with bacterial pathogens (44). Overall, the advantages of memorization, homing, and the immune reconstitution capacity indicate that Tcms might be more persistent and effective than CIK cells in HCC patients. Therefore, our study aimed to explore the efficacy and safety of Tcm transfusion combined with TACE in postoperative HCC patients with MVI, which has not been reported in previous studies.

## Materials and Methods

Additional information is provided in **Supplementary File S1** (**Trial registration number** NCT03575806).

### Patient Selection

A total of 52 HCC patients who underwent curative hepatectomy at the Department of Hepatobiliary Surgery, Cancer Hospital of Chinese Academy of Medical Sciences between October 2016 and September 2018 were enrolled in this study. All subjects met the following criteria: (1) pathological diagnosis of primary HCC with microvascular invasion; (2) R0 tumor resection; (3) Child-Pugh grade A; (4) Eastern Cooperative Oncology Group (ECOG) performance status score of 0; (5) adequate liver, kidney, and marrow functions based on routine blood tests; (6) qualified radiography data within 4 weeks (28 ± 7 days) after enrollment; and (7) no evidence of recurrent HCC or PVTT. The present study was carried out in accordance with the Declaration of Helsinki revised in 1983. The prospective study was approved by the Committee on Medical Ethics of the Cancer Hospital of Chinese Academy of Medical Sciences.

This study did not employ a randomized design, and patients were recruited according to their willingness to receive Tcm transfusion as adjuvant therapy after being screened and meeting the eligibility criteria. Patients who consented to participate in the trial and underwent Tcm infusion combined with TACE were assigned to the Tcm group. Patients who refused Tcm infusion were assigned to the control group and received only TACE. The patients in both groups underwent anatomical hepatectomy or nonanatomical hepatectomy, as decided by the principal investigator. The tumor size and resection margin were measured before specimen fixation.

Both MVI and histological differentiation were examined by microscopy. We evaluated the degree of MVI according to the following three risk factors based on all sections in each case: the number of invaded vessels (≤5 and >5); the number of invading carcinoma cells (≤50 and >50); and the distance of invasion from the tumor edge (≤1 and >1 cm). Cases with no risk factors were classified as M0; those with one risk factor were classified as M1; and those with two or three risk factors as M2 (45).

### Therapeutic Regimens

#### TACE

At 1 month after curative hepatectomy, after examination by radiography (enhanced computed tomography (CT)/magnetic resonance imaging (MRI)) and determination of liver function recovery, a hepatic arterial catheter was placed into the proper hepatic artery through the femoral artery using the Seldinger technique. A mixed emulsion of fluorouracil (1.0 g), adriamycin (40 mg), cisplatin (50 mg), and lipiodol (10–20 ml) was infused into the remnant liver *via* the catheter.

#### Tcm Therapy

**Figure 1:**
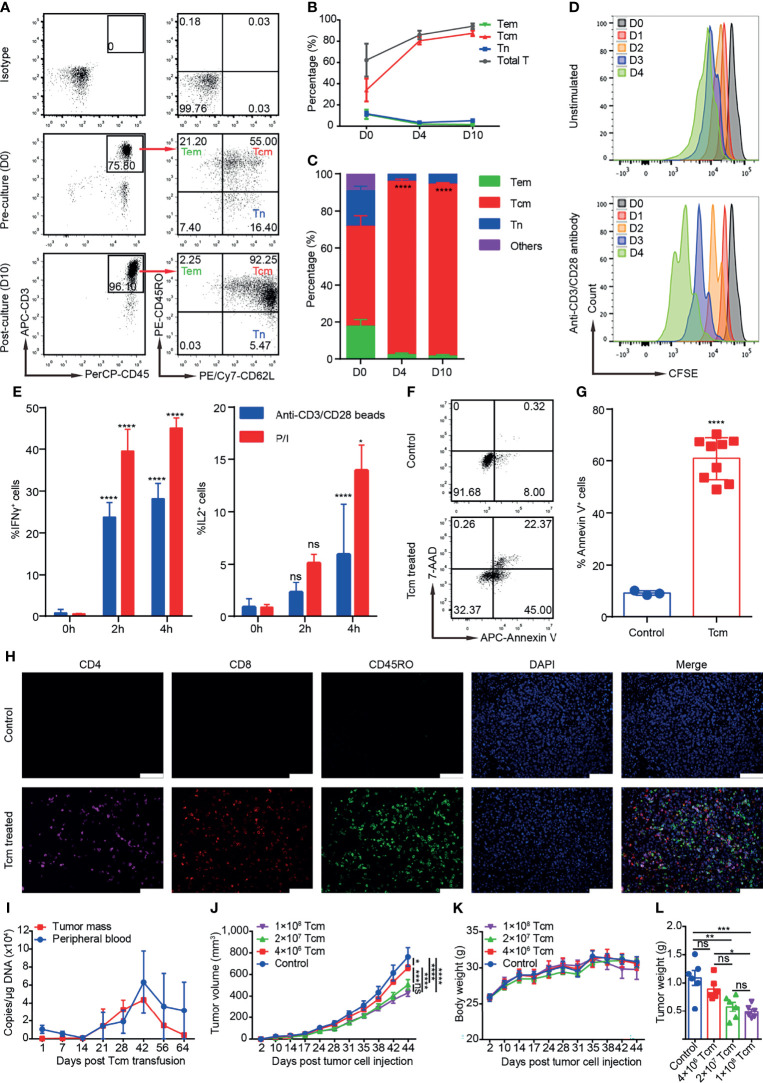
Phenotypes, cytotoxicity, and safety profiles of Tcms in the preclinical study, which was performed as described in the **Supplementary Methods**. **(A)** Representative flow plots of Tcm (central memory T cell), Tem (effector memory T cell), and Tn (naïve T cell) surface marker expression in cultured monocytes on D0 and D10. **(B)** Percentage of the indicated T-cell subsets among monocytes cultured from D0 to D10. Data are shown as the mean ± SD (*n* = 3). **(C)** Percentage of Tcms, Tems, and Tns in the CD3^+^ T-cell population among monocytes cultured from D0 to D10. Data are shown as the mean ± SD (*n* = 3). **(D)** Proliferation rate of cultured Tcms on D14 stimulated with anti-CD3/CD28 antibody, shown as carboxyfluorescein succinimidyl ester (CFSE) staining and cultured for over 4 consecutive days; unstimulated Tcms are shown as a control. **(E)** Cytokine secretion of cultured Tcms on D14 shown as the percentage of IFNγ^+^ and IL2^+^ cells measured at 0, 2, and 4 h after stimulation with phorbol myristate acetate/ionomycin (P/I) or anti-CD3/CD28 beads. Data are shown as the mean ± SD (*n* = 3). **(F, G)** Cytotoxicity (*in vitro* killing) of cultured Tcms on D14 against human hepatocellular carcinoma cell line QGY-7703, as measured by targeted cell stained with 7-AAD and Annexin V, detected by flow cytometry. Tcms and QGY-7703 cells were coincubated for 6 h at an effector:target ratio of 1:5. Representative flow plots and the percentage of Annexin V^+^ cells in target cells are shown in **(F, G)**, respectively. **(H)** Representative immunohistochemistry-paraffin (IHC-P) images of tumor tissue slices immunostained with antibodies against CD4, CD8, and CD45RO and DAPI. Mice were inoculated subcutaneously with QGY-7703 tumor cells on D0 and then treated with Tcms or PBS (control) by tail vein injection, and on D42, the subcutaneous tumor was stripped for IHC-P. **(I)** Tcm persistence (shown as copies/μg DNA) in the tumor mass and peripheral blood of NPG mouse recipients measured by qPCR at the indicated time after Tcm transfusion by tail vein injection. Data are shown as the mean ± SD (*n* = 6 mice). **(J–L)** Different doses of Tcms were transfused into QGY-7703 tumor-bearing mice, and the **(J)** tumor volume and **(K)** body weight were measured on the indicated days. **(L)** The tumor weight of each mouse was measured on D42 posttumor cell injection. Animals in the control group received only PBS. Data are shown as the mean ± SEM (*n* = 6 mice). ns, not significant; ^*^ *p* < 0.5; ^**^ *p <* 0.01; ^***^ *p* < 0.001; ^****^ *p* < 0.0001.

Two or 3 days before TACE, 80–100 ml of the patient’s venous blood was collected for monocyte extraction by Ficoll-Paque™ PLUS (GE Healthcare, Chicago, IL, USA) according to manufacturer’s protocol. The collected cells were seeded at an initial density of 1–1.5 × 10^6^ cells/ml into a new 75-cm^2^ culture flask, which had been pretreated with anti-CD3 (3 μg/ml, Acro Biosystems, Newark, DE, USA) and anti-CD28 (1 μg/ml, Acro Biosystems) with GT-T551 H3 serum-free medium (TAKARA, Kusatsu, Japan) containing IL-2 (200 IU/ml, Jiangsu Kingsley Pharm, Wuxi, China), IL-7 (5 ng/ml, Novus, St. Louis, MO, USA) and IL-15 (2.5 ng/ml, Novus) at 37℃, 5% CO_2_ in the GMP laboratory. On day 4, the cells were transferred from cell culture flask to cell culture bag and fresh medium was added to the culture on day 4/7/10. Tcm cells were harvested on day 14 and confirmed negative for bacteria, fungi, and mycoplasma; the viability, quantity, and purity of Tcms (CD45RO^+^CD62L^+^CD3^+^/CD45^+^) were measured by qualified methods. In general, 2–5 × 10^9^ cells could be harvested after culture for 14 ± 1 days, and the proportion of Tcms were more than 80% for most patients. The characteristics of Tcms were analyzed in a preclinical study (**Figure 1**), as described in the **Supplementary Methods**; the surface marker expression profiles of cultured Tcms from two selected patients are shown (**Supplementary Figure S1**). For intravenous administration, Tcms harvested on day 14 were resuspended in 100 ml of cool saline with 1% human serum albumin. Blood collection and Tcm transfusion were performed again in the next month, and the procedures were the same as those for the first transfusion. Acute adverse events, such as fever and rash that occurred during cell transfusion were recorded.

### Follow-Up Evaluation and Therapeutic Efficacy Analysis

All enrolled patients were evaluated for recurrence in the first 1 month after hepatectomy by enhanced CT or MRI. The patients were then reevaluated every 3 months by enhanced CT or MRI and determination of the serum alpha-fetoprotein (AFP) level until death or withdrawal from the follow-up program. The recurrence rates reported are based on radiography results.

RFS was defined as the interval (in months) between hepatectomy and the diagnosis of recurrence using either intrahepatic recurrence or extrahepatic metastasis as the primary end point. OS was defined as the interval (in months) from the date of hepatectomy to the date of death. This study was terminated on October 1, 2019, and the last follow-up was considered the end of the study.

### Analysis of Adverse Events and Quality of Life

During Tcm treatment, the patients were followed up every 4 weeks with routine blood tests and liver and kidney function tests for safety monitoring until 4 weeks after the completion of both Tcm transfusion regimens. Thereafter, the patients underwent further routine blood tests and liver and kidney function tests every 12 weeks for 48 weeks or until the last follow-up before October 1, 2019. At every visit, the patients were also requested to complete the Functional Assessment of Cancer Therapy-Hepatobiliary (FACT-Hep) table to evaluate quality-of-life (QOL) (46).

Adverse events resulting from the administration of more than one Tcm transfusion were evaluated using Common Terminology Criteria Adverse Events, version 4.0 (CTCAE v4.0).

### Statistical Analysis

The SPSS 22.0 statistical software package (IBM, Armonk, NY, USA) was used for data analyses. Data are expressed as the mean and range. Quantitative data and percent data were compared using *t*-tests and Chi-square tests, respectively. Discrete variables were compared with Chi-square tests. RFS time is presented by Kaplan-Meier survival curves, and survival analysis was performed using the Gehan-Breslow-Wilcoxon test. Univariate and multivariate Cox proportional hazard models were applied for prognostic risk factor analysis. Seventeen factors associated with RFS after hepatectomy were identified by univariate analysis, and significant factors (*p* < 0.15) were evaluated by multivariate analysis to identify valuable independent factors for predicting RFS. For all tests, *p* < 0.05 was considered statistically significant.

## Results

### Patient Characteristics

From October 2016 to September 2019, we recruited 52 HCC patients with MVI who underwent curative hepatectomy for this study. Patients were excluded from the final analysis if they had metastatic HCC (one patient in the Tcm group), voluntarily withdrew (two patients in the Tcm group), or were ineligible for curative hepatectomy with HCC recurrence within 1 month after hepatectomy (one patient in the Tcm group). Ultimately, 48 patients were enrolled, including 23 in the Tcm group and 25 in the control group, according to their willingness.

The baseline characteristics of all HCC patients with MVI are presented in **Table 1**. Overall, the median age was 53.5 years (range: 27–77 years). Of the 48 HCC patients, 35 (72.9%) were male and 13 (27.1%) female. All patients had an ECOG score of 0 points before hepatectomy. In addition, 37 (68.6%) and 22 (25.0%) patients were positive for hepatitis B surface antigen (HbsAg) and had liver cirrhosis. A total of 22 patients (45.8%) underwent anatomical liver resection, and the average tumor size was 5.51 cm (range: 1.5–11.5 cm). Twenty (41.7%) and 27 (56.3%) patients were diagnosed with poorly and moderately differentiated HCC, respectively, and 33 (65.1%) and 12 (27.9%) were diagnosed with M1 and M2, respectively, according to MVI classification criteria.

**Table 1:** Patient characteristics of the overall cohort.

	Control group (*n* = 25)	Tcm group (*n* = 23)	*P* value
**Age (year)****^a^**	53.4 (27~68)	53.7 (31~77)	0.94
**Sex**			
Male	19 (76)	16 (69.6)	0.62
Female	6 (24)	7 (30.4)	
**ECOG score**			
0	25 (100)	23 (100)	–
1	0 (0)	0 (0)	
Tumor diameter^a^ (cm)	5.98 (1.6~11.5)	5.0 (1.5~11.0)	0.24
Tumor volume^a^ (cm^3^)	96.1 (1.2~390.2)	78.0 (0.8~450.0)	0.60
**BCLC stage**			
0	1 (4)	2 (8.7)	0.32
A	22 (88)	21 (91.3)	
B	2 (8)	0 (0)	
**Tumor differentiation**			
Well	0 (0)	1 (4.3)	0.36
Moderate	16 (64)	11 (47.8)	
Poor	9 (36)	11 (47.8)	
**MVI**			
M0	1 (4.0)	2 (8.7)	0.17
M1	15 (60.0)	18 (78.3)	
M2	9 (36.0)	3 (13.0)	
**HBsAg**			
Positive	21 (84.0)	16 (69.6)	0.06
Negative	2 (8.0)	7 (30.4)	
**HCV**	2 (8.0)	0 (0)	–
**Liver cirrhosis**			
Yes	10 (40.0)	12 (52.2)	0.40
No	15 (60.0)	11 (47.8)	
**Type of resection**			
Anatomical	15 (60.0)	7 (30.4)	0.04
Nonanatomical	10 (40.0)	16 (69.6)	
**Liver function**			
ALT (U/L) ^a^	48.5 (12~230)	29.5 (9~63)	0.09
AST (U/L) ^a^	44.1 (16~202)	32.4 (14~124)	0.25
TBIL (µmol/L)^a^	14.4 (5.8~32.5)	14.3 (2.5~29.4)	0.95
ALB (g/L) ^a^	43.9 (24.2~51.3)	43.3 (26.1~50.5)	0.80
**AFP (ng/ml)****^a^**	1,702.7 (3.12~15,101)	4,369.4 (1.6~50,149)	0.28

a Average (range).

ECOG, Eastern Cooperative Oncology Group; BCLC, Barcelona Clinic Liver Cancer; HCV, hepatitis C virus; ALT, alanine aminotransferase; AST, glutamic oxaloacetic aminotransferase; TBIL, total bilirubin; ALB, albumin; AFP, alpha-fetoprotein.

### Comparison of RFS According to Tcm Treatment

The median follow-up time was 20.5 months (95% CI: 17.05–22.55 months) in all patients, 21.7 months (95% CI: 16.17–24.16 months) in the Tcm group and 18.43 months (95% CI: 15.40–23.54 months) in the control group. No in-hospital death occurred.

**Figure 2:**
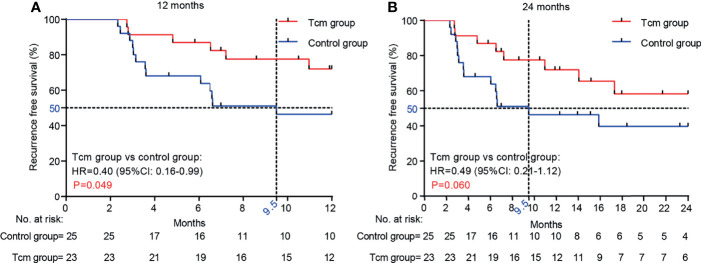
Comparison of RFS between the Tcm group and the control group at **(A)** 12 months (*p* = 0.049) and **(B)** 24 months (*p* = 0.060). *p-*values were calculated with the Gehan-Breslow-Wilcoxon test.

Kaplan-Meier curves and the Gehan-Breslow-Wilcoxon test were used to analyze RFS in the two groups, which was higher in the Tcm group than in the control group (72.0% *vs.* 46.4% for 1 year; 58.18% *vs.* 39.14% for 2 years) (**Figure 2**). In addition, the median RFS time was 9.5 months in the control group, whereas the cutoff date was not reached in the Tcm group because both 12- and 24-month RFS rates were still greater than 50% (**Figure 2**). The Gehan-Breslow-Wilcoxon test revealed a significantly better RFS in the Tcm group when the follow-up duration was 12 months (*p* = 0.049; HR = 0.40; 95% CI: 0.16–0.99), but the difference was not statistically significant at 24 months (*p* = 0.06; HR = 0.49; 95% CI: 0.21–1.12). Overall, Tcm combined with TACE obviously extended the RFS time in the early period after hepatectomy, with protective efficacy possibly lasting for 12 months. However, because the AFP level gradually decreased after hepatectomy (returning to normal in almost all patients within 2 months after hepatectomy) and the AFP level increased upon relapse, the change in the latter was not significant between the twbo groups (**Supplementary Figure S2**).

### Impact of Tcm Therapy on the RFS of Postoperative HCC Patients

In the entire cohort, tumor recurrence developed in 22 (45.8%) patients, including eight (34.8%) in the Tcm group and 14 (56.0%) in the control group. The significant predictors (*p* < 0.15) obtained by univariate analysis, such as Tcm therapy, tumor diameter, and tumor volume, were entered into the multivariate logistic regression model to identify valuable independent predictors for RFS. Nonetheless, no significant independent predictors (*p* < 0.05) for RFS in postoperative HCC patients were found; the *p-*value of Tcm therapy was 0.107 (HR = 2.312, 95% CI: 0.835–6.400). A reasonable explanation is the small sample size and the small difference in tumor diameter and tumor volume at baseline between the two groups. The results of univariate and multivariate analyses for RFS are shown in **Table 2**.

**Table 2:** Univariate and multivariate analyses of prognostic factors for RFS.

	Univariate analysis		Multivariate analysis	
	HR (95% CI)	*p*-value	HR (95% CI)	*p*-value
Tcm treatment	2.502 (0.948~6.60)	0.064	2.312 (0.835~6.400)	0.107
Age	1.014 (0.974~1.056)	0.490		
Sex (male)	0.383 (0.111~1.318)	0.128		
BCLC (A)	25.553 (0.003~2.017x10^5^)	0.479		
Anatomical resection	0.516 (0.207~1.285)	0.155		
Tumor number	1.993 (0.266~14.941)	0.502		
Tumor diameter	1.134 (0.970~1.325)	0.114	0.998 (0.725~1.375)	0.992
Tumor volume	1.003 (0.999~1.006)	0.105	1.002 (0.995~1.010)	0.511
Differentiation (poor)	0.773 (0.492~1.213)	0.262		
MVI (M1)	0.752 (0.282~2.004)	0.568		
HBsAg (positive)	0.918 (0.304~2.774)	0.880		
Cirrhosis	0.931 (0.378~2.292)	0.877		
ALT	1.002 (0.991~1.013)	0.745		
AST	0.997 (0.982~1.012)	0.652		
TBIL	0.975 (0.908~1.046)	0.478		
ALB	1.027 (0.948~1.113)	0.510		
AFP	1.000 (1.000~1.000)	0.617		

ALT, alanine aminotransferase; AST, glutamic oxaloacetic aminotransferase; TBIL, total bilirubin; ALB, albumin; AFP, alpha-fetoprotein.

### Patient-Reported Outcome (FACT-Hep) Analyses

**Figure 3:**
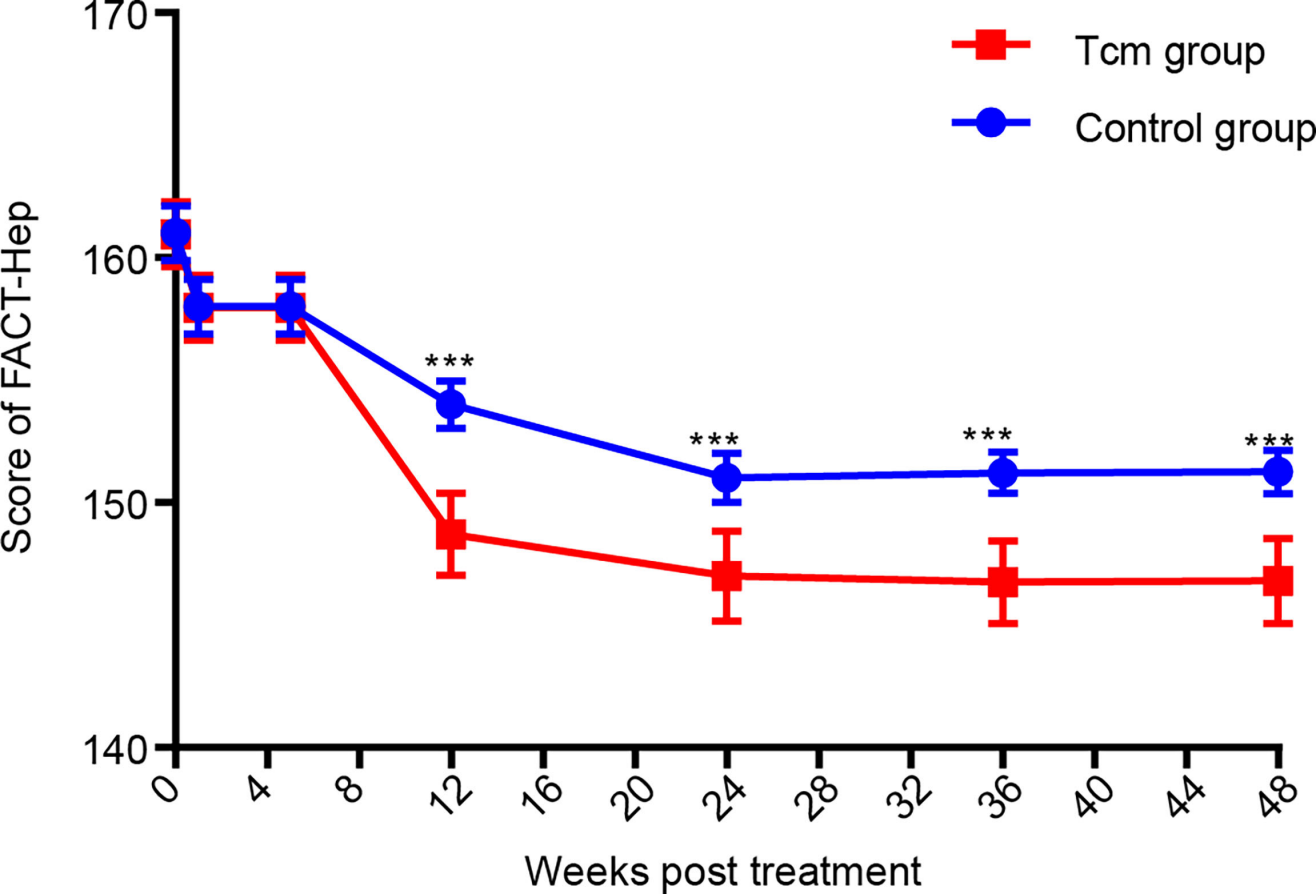
Comparison of FACT-Hep scores according to Tcm treatment. ^***^ *p* < 0.001.

Functional Assessment of Cancer Therapy-General (FACT-G) contains 27 items for assessing four main domains: physical wellbeing, social and family well-being (range = 0–28), emotional well-being (range = 0–24), and functional well-being (range = 0–28). The scores for the FACT-G and the hepatobiliary cancer subscale (HCS), including 18 items (range = 0–72) for assessing specific concerns and issues in patients with HCC, were summed to obtain the FACT-Hep total score, which ranged from 0 to 180. High scores for all FACT-Hep dimensions are interpreted as high QOL (47). After collecting all patient FACT-Hep scores during the 48-week follow-up program, the data showed that the QOL of patients in the Tcm group was obviously better than that of those in the control group (**Figure 3**). In detail, the subscores of the FACT-G and HCS at the time of the 2nd Tcm transfusion and at 4 weeks after completing the 2nd Tcm transfusion demonstrated that Tcm transfusion mainly reduced the median HCS score (61.0 *vs.* 65.0 and 54.1 *vs.* 63.1, see **Supplementary Table S1**). It was concluded that the antitumor efficacy of Tcm treatment can relieve symptoms due to HCC as well as liver function injury.

### Analysis of Adverse Events After Tcm Therapy

**Figure 4:**
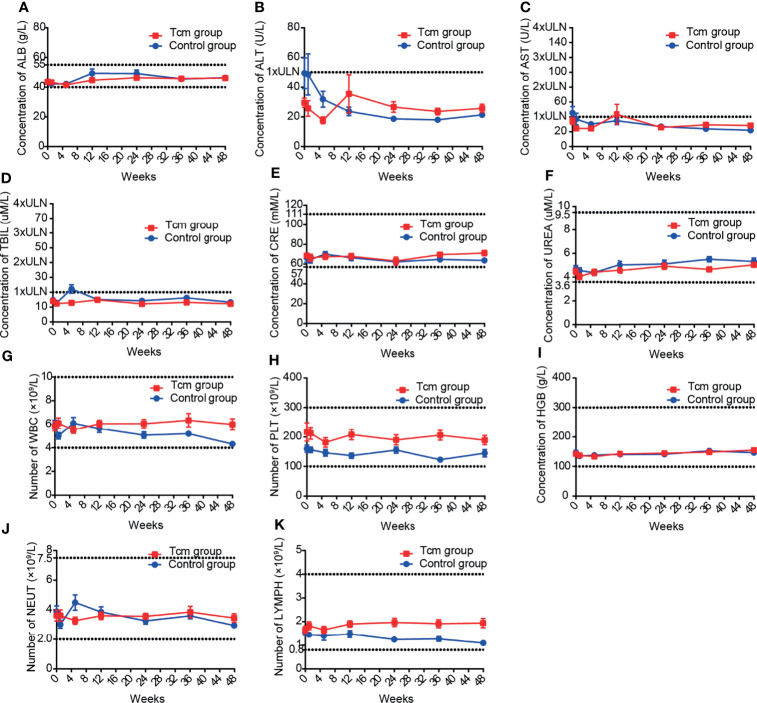
Mean laboratory results of **(A–F)** liver and kidney function tests and **(G–K)** blood tests in the Tcm group and control group during the follow-up period. The upper and lower dotted lines in each graph are the upper and lower limits of normal, respectively; for graph with only one dotted line, the lower limits of normal is 0. ( ALB, albumin; ALT, alanine aminotransferase; AST, glutamic oxaloacetic aminotransferase; TBIL, total bilirubin; CRE, creatinine; UREA, urea; WBC, white blood cell; PLT, platelet; HGB, hemoglobin; NEU, neutrophil; LYMPH, lymphocyte; ULN, upper limit of normal).

Twenty-six patients administered more than one Tcm transfusion and 25 patients in the control group were included in safety analysis, and the main clinical symptom after Tcm treatment was transient fever (incidence rate: 3.08%). The results of blood tests and liver and kidney function tests for all patients were collected every 4 weeks during Tcm treatment and every 12 weeks after Tcm treatment for 48 weeks or the last follow-up before October 1, 2019. The most common adverse reaction was an increase in bilirubin, occurring in 17.32% of patients in the Tcm group and in 25.56% of patients in the control group, indicating that bilirubin elevation might be caused by hepatectomy and not Tcm treatment. In addition, no grade 3 or 4 adverse events or deaths occurred in either group. After statistical calculation of other test indices, mean aminotransferase, albumin, urea, creatinine, platelet, hemoglobin, white blood cell lymphocyte, and neutrophil results were all within normal limits (**Figure 4**), and nearly no difference in the incidence of abnormal results was found between the groups (**Table 3**). Nonetheless, we did find that only 3.15% of patients in the Tcm group but that 11.11% of patients in the control group experienced a decrease in the number of lymphocytes, indicating that Tcm transfusion efficiently relieved lymphocyte deficiency in postoperative HCC patients.

**Table 3:** Statistics of adverse events in the control and Tcm groups.

	Control group				Tcm group			
	Increase		Decrease		Increase		Decrease	
	Frequency	Incidence	Frequency	Incidence	Frequency	Incidence	Frequency	Incidence
ALT	8	8.89%	–	–	5	3.94%	–	–
AST	10	11.11%	–	–	3	2.36%	–	–
TBIL	23	25.56%	–	–	22	17.32%	–	–
ALB	1	1.11%	1	1.11%	0	0	–	–
UREA	1	1.11%	–	–	1	0.79%	–	–
CRE	2	2.22%	–	–	1	0.79%	–	–
PLT	0	0	1	1.11%	6	4.72%	4	3.15%
HGB	0	0	0	0	0	0	0	0
WBC	1	1.11%	5	5.56%	0	0	6	4.72%
NEUT	0	0	3	3.33%	0	0	6	4.72%
LYMPH	0	0	10	11.11%	0	0	4	3.15%

ALT, alanine aminotransferase; AST, glutamic oxaloacetic aminotransferase; TBIL, total bilirubin; ALB, albumin; UREA, urea; CRE, creatinine; PLT, platelet; HGB, hemoglobin; WBC, white blood cell; NEU, neutrophil; LYMPH, lymphocyte.

## Discussion

MVI is an important independent predictor of recurrent HCC after hepatectomy (15, 16), and pa-TACE is recommended for adjuvant treatment to prevent recurrence in HCC patients with MVI after hepatectomy (23). Regardless, the limited and controversial clinical benefits of pa-TACE necessitate improvement of this therapy, and combined therapy is an approach worth considering.

The pathogenesis of MVI is closely related to the immunosuppressive tumor microenvironment, in which tumor-infiltrating lymphocytes display characteristics of exhaustion. Adoptive cell therapies, including autologous transfer of lymphokine-activated killer (LAK) cells (48), tumor-infiltrating lymphocytes (49) and CIK cells (32), have exhibited antitumor effects in preventing the postoperative recurrence of HCC. However, their clinical effects are limited due to short-term *in vivo* persistence, reliance on multiple cytokine boosts and a lack of focused analysis involving HCC patients with MVI. For example, at least four CIK transfusions are required to prevent short-term recurrence in postoperative HCC patients (32, 35). In contrast, the potent ability of Tcms for rapid activation, self-renewal, lymphatic homing and immunological reconstruction confers them with the capacity for long-term retention *in vivo* with strong antitumor activity (40). Indeed, our study was the first to find that Tcm treatment combined with TACE significantly prolonged the median RFS time compared with TACE treatment alone (>24 *vs.* 9.5 months) and largely improved patient-reported outcomes. In addition, Tcm treatment did not cause obvious damage in terms of liver or kidney function, blood indices, or systemic response markers, even in HCC patients with reduced liver function. Overall, the liver function of patients who incurred previous damage recovered almost to normal by 2–3 weeks after completing Tcm treatment, which indicates the safety of this treatment. The failure of this study to identify Tcm treatment as an independent prognostic factor associated with HCC recurrence might be due to the small sample size, nonrandomized design, short follow-up period, and/or single-centered nature, and further investigations are needed to demonstrate efficacy.

In conclusion, this pilot study for the first time expands the indications for Tcm treatment combined with TACE as an adjuvant therapy in postoperative HCC patients with MVI. The clinical outcome is encouraging but still speculative for limited number of patients and short follow-up period. Additional phase II studies should be performed to evaluate the efficacy of this treatment on more HCC patients. Longer follow-up period (4–5 years) may be warranted to explore whether this treatment could improve OS of postoperative HCC patients with MVI. Also, examination of cytokine profile (IL-6, IFN-γ, IL-10, TGF-β) and HCC-associated antigen-reactive lymphocyte populations could be performed to obtain more information to optimize the treatment formula. In all, further clinical trials are needed to test the efficacy of Tcm treatment and to identify the most suitable patient population.

## Data Availability Statement

The raw data supporting the conclusions of this article will be made available by the authors, without undue reservation.

## Ethics Statement

The studies involving human participants were reviewed and approved by the Committee on Medical Ethics of the Cancer Hospital of Chinese Academy of Medical Sciences. The patients/participants provided their written informed consent to participate in this study.

## Author Contributions

HZ, JC, and JM contributed to the study design. HZ, JC, and JZ contributed to patient enrollment and study management. HZ, JM, DL and HX contributed to data analysis. JM, HX, DL and HZ wrote the manuscript. All authors contributed to data interpretation and planning and critical review of the manuscript content. All authors were responsible for the final decision to submit.

## Funding

This study was sponsored by Newish Technology (Beijing) Co. Ltd, S&T program of Hebei (program No. 20372403D), National Science and Technology Major Projects for Major New Drugs Innovation and Develop (grant No. 2018ZX09711003-004-002), the National Natural Science Foundation of China (81972311), the Non-profit Central Research Institution Fund of Chinese Academy of Medical Sciences (2019PT310026), and Sanming Project of Medicine in Shenzhen (No. SZSM202011010). Tcm treatment was sponsored by Newish Technology (Beijing) Co. Ltd.

## Conflict of Interest

DL and HQ are current employees of Newish Technology (Beijing) Co., LTD.

The authors declare that this study received funding from Newish Technology Co. LTD. The funder had the following involvement in the study: DL and HQ were involved in the preclinical study design, collection, analysis, interpretation of the preclinical data and the revision of the manuscript. Tcm cells were manufactured in the GMP laboratory of Newish Technology.

The remaining authors declare that the research was conducted in the absence of any commercial or financial relationships that could be construed as a potential conflict of interest.

## Publisher’s Note

All claims expressed in this article are solely those of the authors and do not necessarily represent those of their affiliated organizations, or those of the publisher, the editors and the reviewers. Any product that may be evaluated in this article, or claim that may be made by its manufacturer, is not guaranteed or endorsed by the publisher.
