# Supplementary material for: Efficacy and Safety of Central Memory T Cells Combined With Adjuvant Therapy to Prevent Recurrence of Hepatocellular Carcinoma With Microvascular Invasion: A Pilot Study
Source: Front Oncol. 2021 Dec 3;11:781029. doi: 10.3389/fonc.2021.781029 (PMC8679661; doi:10.3389/fonc.2021.781029)
Supplement: Supplementary file 1 [file DataSheet_1.pdf]

Supplemental material

Supplementary methods

Cell lines

The human hepatoma cell line QGY-7703 was cultured with RPMI 1640 plus 10% FBS at 37°C and 5% CO_2 and passaged after trypsin digestion.

Animals

Female NPG mice were purchased from Vitalriver (Beijing) for pharmaceutical kinetics measurement of transfused Tcm conducted by Joinn-lab (Beijing). Male NCG mice were purchased from GemPharmatech (Jiangsu, China) for Tcm *in vivo* antitumor activity evaluation conducted by Yicon (Beijing) Medical Science And Technology Co., Ltd.

Flow cytometry

Cells cultured on the indicated days were harvested in tubes and stained with the indicated antibodies according to the manufacturer's protocol. Anti-human CD45, CD3, CD45RO, CD62L, CD4 or CD8 antibodies were purchased from Biolegend. The stained samples were detected using a BD Canto II cytometer and analyzed by FlowJo software.

Proliferation assay

Tcm cells were suspended at 1 $\times 10^7$ cells/mL in PBS, followed by CFSE reagent addition and incubation in the dark for 20 min. These cells were seeded into anti-CD3/CD28-coated plates at 1.5×10^6 cells/mL cultured with RPMI 1640 plus 10% FBS. CFSE staining of the cells was analyzed by flow cytometry on the indicated days.

Cytokine production detection

Cultured Tcm cells were stimulated with anti-CD3/CD28 antibody (2 μg/mL) and PMA (50 ng/mL)/inonomycin (500 ng/mL) for 0, 2, and 4 h at 37°C and 5% CO_2. The cells were collected at the indicated times, fixed with a BD fix/permeation kit, and stained with anti-IFNγ and anti-IL2 antibodies. The stained samples were analyzed using a BD Canto II cytometer**.**

In vitro *cytotoxicity assay*

Cultured Tcm cells were incubated with the tumor cell line QGY-7703 for 6 h at an effector:target ratio of 1:5 in RPMI 1640 plus 10% FBS 37°C and 5% CO_2. The suspension was removed; the attached cells were digested, collected and stained with 7-AAD and anti-Annexin V antibody according to the product manuals. Cytotoxicity is displayed as the percentage of the Annexin V^+ cell population among all tumor cells.

Subcutaneous tumor cell implantation and tumor measurement

For Tcm *in vivo* antitumor activity evaluation, 1×10^6 QGY-7703 tumor cells were resuspended in 100 μL PBS and injected subcutaneously into each NCG mouse. Tcms were transfused intravenously on day 2 post tumor cell implantation at dosages of 4×10^6, 2×10^7 and 1×10^8 cells per mouse. The tumor volume and body weight were measured on the indicated days. The calculation formula was tumor volume= 0.5 × length × width^2. On day 42 post tumor cell implantation, the mice were sacrificed, and the tumor mass was measured.

Surgical resection of the QGY-7703 tumor mass and immunohistochemistry-paraffin (IHC-P)

On day 42 post tumor cell implantation, the tumor mass was surgically removed by using a biopsy punch. The samples were fixed with ethanol/xylene and embedded in paraffin wax. Three-micrometer-deep slices were prepared from paraffin-embedded tissues using a slicing machine (Leica) and attached to glass slides. The tissue sections were dewaxed and hydrated with xylene and gradient alcohol and washed with distilled water for 5 min; for antigen retrieval, the sections were placed in repair solution containing citric acid aqueous solution (pH 6.0), heated at 100°C for 2.5 min, and cooled to room temperature. Next, the samples were washed with PBS 3 times for 3 min; peroxidase blocker was added and incubated at room temperature for 10 min. The samples were washed with PBS 3 times for 3 min, the sealing liquid was added and incubated at room temperature for 60 min, and appropriate dilutions of CD45RO, CD8 and CD4 were added and incubated overnight at 4 °C. Washing with PBS was performed 3 times for 3 min. Fluorescently labelled anti-rabbit IgG and anti-mouse IgG were added and incubated are room temperature for 30 min. After washing with PBS three times for 3 minutes, DAPI solution was added and incubated for 10 min at room temperature away from light. The stained samples were sealed and observed under a microscope. This experiment was conducted by Yicon (Beijing) Medical Science And Technology Co., Ltd.

Detection of transfused Tcm* in vivo *by qPCR

Tcms were injected intravenously into NPG mice bearing a QGY-7703 tumor mass (ranging from 0~10.66 mm^3) at a dosage of 3×10^9 cells/kg per mouse. The presence of Tcm in recipient peripheral blood and tumor mass were detected by qPCR method developed by Joinn-lab (Beijing), with 40 copies/μg DNA as the threshold.

Supplementary figures and tables

Blank:

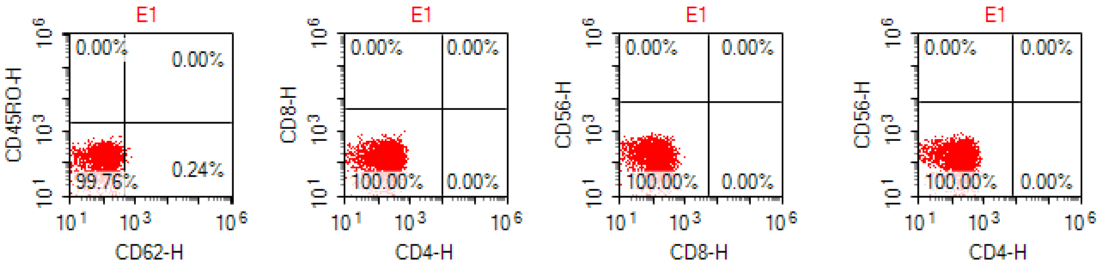

HCC Patient 1:

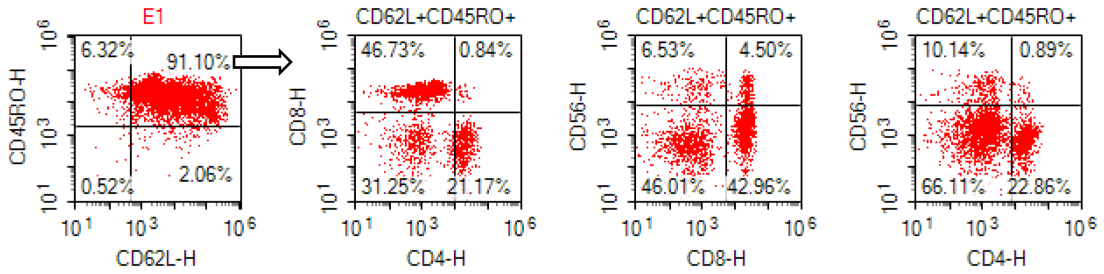

HCC Patient 2:

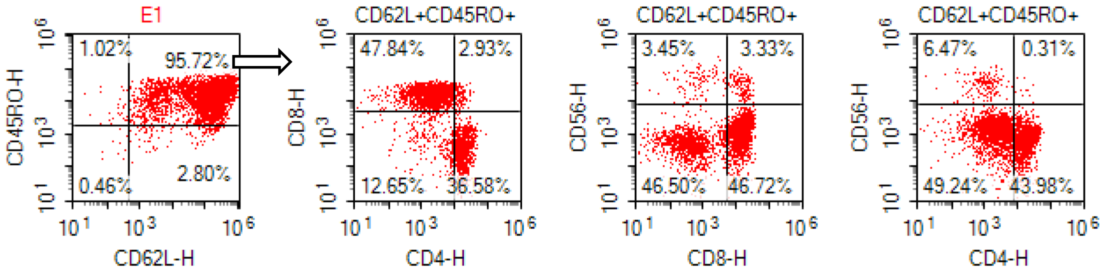

Supplementary figure 1 Flow plots showing surface marker expression of cultured Tcm cells ($CD45RO^{+}CD62\ L^{+}CD4^{+}CD56^{-}$ or $CD45RO^{+}CD62\ L^{+}CD8^{+}CD56^{-}$) from two different HCC patients.

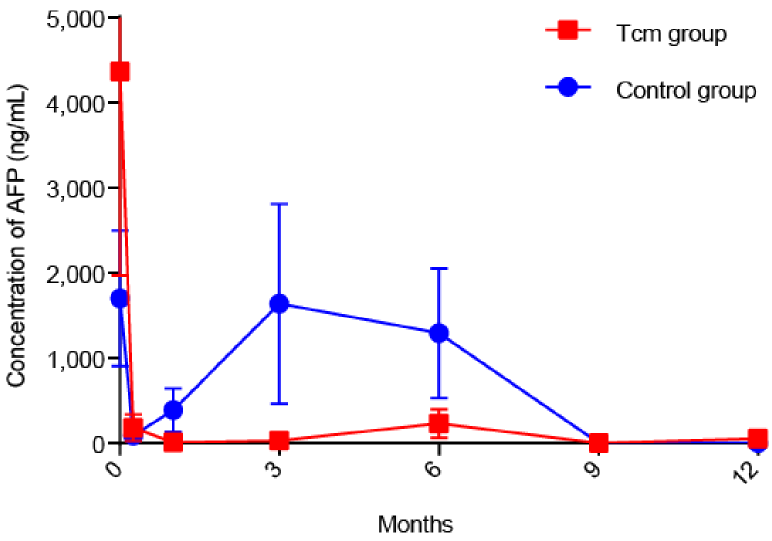

Supplementary figure 2 Serum AFP levels in the Tcm group and control group at the 1-year follow-up. There was no significant difference between the two groups.

Supplementary table 1 Subscores of FACT-Hep in the control group and Tcm group at the 5th week and 12nd week. #: P<0.001

5th week	Score		12nd week	Score	
	Control group	Tcm group		Control group	Tcm group
physical	25.0	24.6	physical	22.9	22.1#
social and family	23.0	23.0	social and family	23.0	23.0
emotional	22.0	22.0	emotional	22.0	22.0
functional	23.0	27.0	functional	23.0	27.0
HCS	65.0	61.0	HCS	63.1	54.1#
Overall	158.0	157.6	Overall	154.0	148.3#
